# Lesbian womens’ access to healthcare, experiences with and expectations towards GPs in German primary care

**DOI:** 10.1186/s12875-016-0562-4

**Published:** 2016-11-21

**Authors:** Oliver Hirsch, Karina Löltgen, Annette Becker

**Affiliations:** Department of General Practice/Family Medicine, Philipps University Marburg, Karl-von-Frisch-Str. 4, Marburg, 35043 Germany

**Keywords:** Female homosexuality, Health surveys, Internet, Questionnaires, Primary health care

## Abstract

**Background:**

Lesbian women have higher rates of physical and psychiatric disorders associated with experiences of discrimination, homophobia and difficulties with coming out. Therefore, easy access to specialized healthcare in an open atmosphere is needed. We aimed to describe women’s access to and experiences with healthcare in Germany, and to assess the responsibility of the general practitioner (GP) compared to other specialities providing primary health care.

**Methods:**

A questionnaire study was conducted via internet and paper-based sampling. Using current literature, we designed a questionnaire consisting of sociodemographic data, sexual orientation, access to care and reasons for encounter, disclosure of sexual orientation, experience with the German health system (discrimination, homophobia), and psychological burden. Depression was assessed using the depression screening from the Patient Health Questionnaire (PHQ-2).

**Results:**

We obtained responses from 766 lesbian women. Although 89% had a primary care physician, only 40% had revealed their sexual orientation to their doctor. The main medical contacts were GPs (66%), gynaecologists (10%) or psychiatrists (6%). Twenty-three percent claimed they were unable to find a primary care physician. Another 12.4% had experienced discrimination. Younger lesbian women with higher education levels and who were less likely to be out to other physicians were more likely to disclose their sexual orientation to their primary care physician. GPs play an important role in healthcare for lesbian women, even in a non-gatekeeping healthcare system like Germany. Study participants suggested improvements regarding gender neutral language, flyers on homosexuality in waiting areas, involvement of partners, training of physicians, directories of homosexual physicians and labelling as a lesbian-friendly practice.

**Conclusions:**

GPs should create an open atmosphere and acquire the respective knowledge to provide adequate treatment. Caring for marginal groups should be incorporated in medical training and further education. Ideally, physicians address patients’ sexual orientation pro-actively in order to address individual needs accordingly.

**Electronic supplementary material:**

The online version of this article (doi:10.1186/s12875-016-0562-4) contains supplementary material, which is available to authorized users.

## Background

Several studies have shown that lesbian women have a higher rate of physical and psychiatric disorders than heterosexual women. Authors have found an increased risk for cardiovascular disorders and cancer, compared to non-lesbian women [1–3], and also for mood disorders, anxiety disorders, substance use and self-injuring or suicidal behaviour [4, 5]. This seems to be associated with minority stress based on discrimination, homophobia and difficulties with coming out [6, 7]. In a large survey at 33 health care sites in the USA, Koh and Ross found that lesbians who were not out were 2–2.5 times more likely to have had suicidal thoughts during the past year. A suicide attempt occurred more often in lesbians and bisexual women who were not out compared to heterosexual women [8].

The aforementioned studies highlight the need for improvements in healthcare provision for lesbian women, especially in primary care, which is usually the first area of contact with the healthcare system. Several factors have been shown to positively influence healthcare provision for these women: The more lesbian women are comfortable with their sexual identity, the better the healthcare provision. This may be because disclosure in the medical context seems much easier for people confident about their sexual identity. Furthermore, the better the integration in their social environment, the more comprehensive their coming out, which leads to a higher openness to physicians who in turn are then better able to adapt to their needs [9].

Another important point is the physicians’ behaviour during the consultation. Physicians have no special qualification for treating homosexual patients. Discrimination, homophobia, and lack of attention regarding gender neutral language have been observed [10, 11]. Recommendations for different medical contexts like primary care, mental health, oncology, and public health are available [4, 12–16]. However, there is still a lack of training resources [16], and the special needs of LGBT (Lesbian, Gay, Bisexual, Transgender) patients are neglected worldwide. On the one hand, GPs and medical students in Ireland do not feel prepared to deal with sexual health issues in lesbian, gay and bisexual patients because they do not possess the required communication skills to competently interact with non-heterosexual patients, as shown in a qualitative study by Stott et al. [17]. On the other hand, LGBT patients want their health providers to be more aware of their health needs. Lesbian and bisexual women in the Cape Town area of South Africa experienced verbal discrimination, especially in public care [18]. In another qualitative study undertaken in Portugal, lesbian women also expressed their demand for a better training of healthcare professionals regarding homosexual issues. Participants suggested that healthcare providers should not show signs of disapproval when a lesbian woman outs herself, they should not assume that all women are heterosexual, and they should provide interventions tailored to the specific needs and interests of lesbian women. The results of the study further showed that a guarantee of confidentiality would have encouraged the interviewed women to disclose their sexual orientation to their physician [19].

The aforementioned requests and conclusions highlight that there are special healthcare needs among lesbian women, but appropriate supportive care is not the norm. Current research worldwide suggests that lesbian women’s particular health needs are neglected, so the question arises whether the same holds true in Germany. Studies on lesbian health in Germany are rare. Most of the available data originates from the USA, where the healthcare system is fundamentally different and therefore not necessarily comparable. It is estimated that between 0.4 and 1.3% of German women are exclusively homosexual [20]. The German Federal Statistical Office mentions in its 2010 microcensus that 27 000 women live together in same-sex partnerships, 10 000 of which are officially registered.

In Germany, primary care physicians are not gatekeepers. Patients have direct access to primary and secondary care specialists, with approximately half of all office-based physicians being GPs. The aim of our study was to examine the lesbian women’s preferred access paths to medical care (GP or specialist) in order to clarify GPs role in caring for these women. We assessed lesbian women’s experiences with and desires regarding medical providers. The questions are: Who are lesbian women’s preferred contact persons in the non-gatekeeping health care system of Germany and what are their reasons for encounter? How do lesbian women experience primary care providers (e.g. use of gender neutral language, asking for sexual orientation) and to what extent have they been confronted with discriminating behaviour within the German health care system? What are predictors for women disclosing their sexual orientation towards their GP? What desires or ideas for improvement do lesbian women express in connection with their healthcare provision?

## Methods

### Questionnaire

Using current literature, we designed a questionnaire consisting of sociodemographic data (age, residence, secondary education level, professional education, employment status, monthly income, marital status, living with partner and being a parent or co-parent), sexual orientation, access to care (specialist or GP), health care utilisation during the previous 12 months and reasons for encounter﻿ (﻿see Additional file 1). Furthermore, we included questions on disclosure of sexual orientation, experience with the German health system (discrimination, homophobia), and psychological burden. Some survey questions were developed by the research team, while those examining depression and discrimination experiences drew on existing instruments [21, 22]. Disclosure of sexual orientation in the private context was examined with the question “Have you disclosed your sexual orientation? If yes, to whom?” Possible answers were: “No disclosure; Just to close friends; Just to family of origin; Just to close friends and family of origin; Comprehensive disclosure (family, friends, colleagues)”. We also asked if the participants were out to their main medical contact person (yes/no).

To measure discrimination experiences we included five questions to be answered “yes” or “no”: “Have you ever refrained from a necessary examination or treatment because you were afraid of being discriminated because of your sexual orientation?”, “Have you ever felt discriminated against by physicians, in hospitals or in other areas of the healthcare system because of your sexual orientation?”, “Have you ever been refused an examination or treatment because of your sexual orientation?”, “Have you ever felt that your physician should know about your sexual orientation prior to an examination or treatment, but you did not disclose it for fear of negative consequences?”, and “Did you ever feel the need to talk about your sexual orientation with your medical contact person, but he/she dismissed it?” [23–25].

Depression was assessed using the depression screening from the Patient Health Questionnaire (PHQ-2) consisting of two items on a four point scale (0–3 points) [21, 22]. It has a maximum score of six points and a cut-off value (≥3 points) leads to a suspected diagnosis of a depressive episode. It has been adapted and validated in the German context [22]. The German version has an internal consistency of *α* = .83, a sensitivity of 87% and a specificity of 78% for major depressive disorder, and it proved sensitive to change. Kroenke et al. have also shown the construct and criterion validity of this instrument [21]. The women were asked whether they were impaired because of further psychological symptoms in the past four weeks, for example regarding sudden feelings of fear and panic (yes/no).

Information on suggestions for improvement in connection with health care provision was collected by a multiple choice question with selected answers based on current literature [26, 27]. Women were asked to select four of twelve proposed improvements (e.g. gender neutral language, special training of physicians, flyers on homosexuality in waiting area, labelling as LGBT friendly practice). Besides they had the opportunity for free text responses.

A pilot study with subsequent adaptation of the questionnaire was performed with 18 lesbian women. It consisted of seven pages and took approximately ten minutes to complete.

### Study design

Women had to be 18 years or older and to identify themselves as a lesbian to be included in the study.

To achieve a high probability of external validity we chose different sampling strategies including different strategies of personal recruitment and using the internet. The internet-based sample was recruited via www.lesarion.de, one of the largest Internet information and dating platforms for lesbian women. A thread called ‘Study for lesbian women’ was created on the bulletin board with a link to the questionnaire on https://www.limesurvey.org/de/, a platform for online surveys. A banner advertisement on the homepage of http://de.lesarion.com/ was used to advertise for the study. Potential participants received written information stating that the study aimed to collect data on healthcare provision for lesbian women in Germany and help to improve the latter. By clicking a verification button, women expressed their informed consent and were able to access the questionnaire. Participants received no financial incentive. Data collection lasted for seven days in June 2011 and resulted in 272 participants.

The paper-based sample was recruited in Cologne and Frankfurt and generally relied on snowball sampling. Recruitment took place at the respective Christopher Street Day Celebrations in 2011, at lesbian counselling centres, self-help groups, private gatherings and reading events. The Christopher Street Day is an annual celebration and demonstration held in different cities in Europe in memory of the first uprisings of LGBT people against police assaults in New York City. Participants were personally contacted by one of the authors (CL). They were informed about the study by a leaflet that contained the same study information offered online and were asked to complete the questionnaire after giving their verbal informed consent. Participants received no financial incentive. The data collection lasted from May to September 2011.

The study was approved by the local medical ethics committee of the Department of Medicine at Philipps University Marburg, Germany with reference number 06/11.

### Statistical methods

Descriptive statistics were used to analyse responses to questionnaire items and to describe women’s access to health care (preferred contact person, reason for encounter) as well as experiences within the health care system, and suggestions for further improvement. Associations between certain attributes were examined using Spearman’s rho. We applied logistic regression analysis [28] to predict disclosure to the GP (yes/no) by age, education, time since outing (less than 12 months, 1 to 5 years, more than 5 years), discrimination experience (dichotomous; at least one “yes” response in the five questions on discrimination lead to coding “yes”), further need for provision of medical service because of sexual orientation (yes/no), and comprehensive disclosure to other physicians (yes/no). We decided not to use a stepwise approach since we had no a priori hypotheses about which variables might best separate the groups, and stepwise methods use only data-driven criteria and are therefore regarded as controversial [29]. Several tests and indices were used to assess model fit:—2 log likelihood test, Hosmer-Lemeshow-Test, deviance measure, likelihood ratio tests for predictors; pseudo-R-squares: Cox and Snell, Nagelkerke and McFadden; and correct classification rates [29].

## Results

### Demographic characteristics

We obtained responses from 766 lesbian women living in Germany. They were all German citizens. Altogether, 494 lesbian women participated in the paper-based sample, evenly distributed between the two locations.

The resulting dataset was checked for duplicates to ensure that no one was recruited in both samples. For this, we sorted the dataset according to demographic characteristics and looked for identical patterns. No duplicates were found. In a different publication we were able to show that the paper-based and Internet-based samples did not differ substantially [30]. We asked for the demographic characteristics of age, sexual orientation, residence, secondary education level, professional education, employment status, monthly income, marital status, living with partner and being a parent or co-parent. They are depicted in Table 1.

**Table 1:** Demographic characteristics of our lesbian sample (*n* = 766)

Mean age	32.5 (SD 9.6); range 18–67
Sexual behavior	
exclusively or mainly homosexual	89.9%
equally homo- and heterosexual	7.5%
mainly heterosexual	1.4%
heterosexual	1.2%
Residence	
major town	46.5%
small town	39.2%
countryside	14.3%
Secondary education level	
low	7.1%
medium	31.6%
high	61.3%
Professional education	
apprenticeship	34.3%
school-based	16.6%
university	32.1%
other	5.2%
none	11.8%
Employed	
yes	82.5%
no	17.5%
Monthly income	
< 400 €	9.4%
401–1000 €	18.4%
1001–4000 €	58.2%
> 4000 €	8.2%
none	5.8%
Marital status	
single	42.2%
in relationship	55.5%
divorced/widowed	2.3%
Living with partner	
yes	30.1%
no	69.9%
Parent/co-parent	
yes	13.6%
no	86.4%

The mean age of the sample was 32.5 years (SD 9.6). The vast majority was exclusively or mainly homosexual. Their place of residence was evenly distributed between major and small towns, with a minority living in the countryside. Levels of graduation and professional education were relatively high. The proportion of unemployment among respondents was considerably higher than the unemployment rate in Germany (17.5% vs. 7%). About one third of the sample had a low income of up to 1000 Euro per month. About half of the women were in a relationship, but only one third were living together with their partner. Merely 13.6% were in the position of being a parent or co-parent.

### Lesbian women’s preferences regarding primary care physicians and reasons for encounter

Approximately 68.7% of the interviewed women stated that they discuss results from secondary care (e.g. cardiologist, neurologist, etc.) with their GP. The main medical contact person was the GP in 66.3% of respondents, the gynaecologist in 10.6% and a psychiatrist in 5.5%. Additionally, 12.3% of respondents did not have a main medical contact person and were not actively looking for one; 2.0% named other medical facilities, and 3.3% provided no data. A female physician was favoured by 26.4%, 69.3% had no preference, 3.9% favoured a male physician, and 0.4% did not answer this question. Twenty-two point eight percent were actively looking for a main medical contact person but were unable to find one. A slightly larger proportion of respondents (45.4%) were out to their gynaecologist compared to their GP (39.2%). In the previous 12 months, most of the women consulted a physician because of an acute illness (76.6%), 50.5% for preventive measures, 22.7% because of psychological symptoms, 20.5% because of a chronic disease, and 4.8% for counselling, e.g. to quit smoking. Over-the-counter drugs were taken regularly by 8.7%, prescription drugs by 25.8%. A majority of the women has regular contact with their GP (74.4%), 54.0% have regular contact with a gynaecologist, 21.0% with a psychiatrist and 29.1% with other physicians. Preventive medical check-ups were attended by 83.6%; details are listed in Table 2.

**Table 2:** Percentages of lesbian women attending preventive medical check-ups

Medical faculty	%
gynaecology	
(at least one preventive check-up)	74.1
mammography	13.7
pap smear	56.1
dentist	76.8
vaccination	46.2
colon cancer screening	5.7

### Women’s experiences within the healthcare system

Only a minority of lesbian women in this study (5.2%) reported being directly asked about sexual orientation by a healthcare provider. Knowledge regarding lesbian-specific issues in physicians was judged positively by 21.5% of lesbian women, 14.5% were satisfied but saw room for improvement, 9.8% said that only basic knowledge was available, 3.1% stated that no lesbian specific knowledge was present, 50.5% said that they were unable to rate this knowledge, and 0.5% did not answer this question.

Not having attended a necessary examination or treatment because of feared discrimination was reported by 7.7%, 12.4% had experienced discrimination in the German healthcare system, 3.8% were denied an examination or treatment because of their sexual orientation, 11.9% did not disclose their sexual orientation to physicians although it was important for examination or treatment, and 16.4% felt the need to talk about their sexual orientation and were not taken seriously by medical personnel. It is well-established that discrimination experience is associated with depressive symptoms [31]. In our sample, discrimination experience was not significantly correlated to depression (PHQ-2; rho = .01, *p* = .82). In our sample, 7.7% reported clinically-relevant depressive symptoms, and 17.1% mentioned that they had experienced a panic attack in the previous four weeks. The mean score in the PHQ-2 (1.4, SD 1.4) was not different from a healthy control group [22].

### Predictors of disclosure of sexual orientation towards the GP

In our sample, 87.9% reported having a primary care provider (resident specialist or GP); however, 60.6% of these respondents had not informed their primary care provider about their sexual orientation. Altogether, 47.8% stated that their main medical contact person was informed about their sexual orientation. For those claiming to have a GP as primary care physician we used logistic regression to predict disclosure to the GP (yes/no) as there is evidence that such a disclosure had a positive impact on healthcare provision for lesbian women. No multicollinearity was present as all variance inflation factors were around 1 and therefore much lower than the critical cut-off of 10 [29]. The likelihood ratio test resulted in three significant predictors after adjusting for multiple testing (*p* = .05/6 = .008) as a test is performed for each predictor separately [28]: age, education and disclosure to other physicians (Table 3). Those with a younger age, a higher formal education and who were not out to other physicians were more likely to have disclosed their sexual orientation to their GP. The overall correct classification rate was 70.9% and consequently unsatisfactory. Compared to the intercept-only model, our model demonstrated a significant improvement in the—2 log likelihood test (*χ* ^2^ (df = 6) = 165.62, *p* < .001). The Pearson deviance measure, as a goodness-of-fit statistic comparing observed with expected frequencies, showed a good fit (*χ* ^2^ (df = 472) = 463.99, *p* = .60). The Hosmer-Lemeshow-Test which is based on a similar approach also shows a good model fit (*χ* ^2^ (df = 8) = 3.25, *p* = .92). The following effect size values occurred: McFadden’s ρ^2^ = .18, Cox and Snell Pseudo-R^2^ = .20, and Nagelkerke Pseudo-R^2^ = .27. McFadden’s *ρ* ^2^ was just under the recommended cut-off of .2 [29], while the other two effect sizes signaled acceptable proportions of explained variance.

**Table 3:** Predictors of disclosure of sexual orientation to primary care physicians: Results of the logistic regression analysis

	B	Likelihood Ratio Test *χ* ^2^ (df = 1)	Significance *p*	Odds Ratio (95% confidence interval)
Disclosure to other physicians	−1.83	92.24	**<.001**	0.16 (0.11–0.23)
Age	−.03	10.62	**.001**	0.97 (0.95–0.99)
Education	.40	8.83	**.003**	1.50 (1.15–1.96)
Time since outing	−.36	5.00	.024	0.70 (0.51–0.96)
Further need of medical service	.15	0.59	.44	1.16 (0.80–1.68)
Discrimination experience	.09	0.22	.64	1.09 (0.76–1.57)

Significant predictors after Bonferroni correction printed in bold

### Areas for improvement

Lesbian women in our study were asked to consider ways to improve interactions with health professionals. These are depicted in Table 4.

**Table 4:** Percentages of lesbian women proposing specific improvements

Area	%
gender neutral language	36.7
flyers on homosexuality in waiting area	29.8
involvement of partner	29.4
training of physicians	27.4
directory of homosexual physicians	24.7
labelling as lesbian friendly practice	24.4
more homosexual physicians	18.8
direct question regarding sexual orientation	16.4
support disclosure	14.9
provide lesbian specific information	14.8
consultation hours for lesbians	8.7
physician should ask regarding social integration	8.2

Unfulfilled global healthcare needs were expressed by 25.6%, 9.5% had general medical requests, 2.6% would like to get access to lesbian information centres, 3.8% would like to receive more information on sexually transmitted diseases, and 12.9% would like to discuss their desire for children. A request for special care because of their sexual orientation was expressed by 30.9%.

## Discussion

Given the evidence of an increased morbidity of lesbian women compared to their heterosexual counterparts [1–4], we conducted a survey on lesbian women’s access to German healthcare to learn about their experiences with and expectations towards primary care physicians. The aim of the study was to clarify the GPs’ role in caring for homosexual women in a non-gatekeeping healthcare system. We received responses from 766 women. The majority of women had regular contacts with their GP and gynaecologist, and two-thirds of them named the primary care physician as their main medical contact person from whom they seek care for acute and chronic illnesses, and discuss results from secondary care, and for non-gynaecological prevention. More than 20% of women had looked for a trustworthy person in health matters without finding one. Experiences or fear of discrimination were reported by 12% of the women. Disclosure of sexual orientation was not pursued by 40% of women, 12% of whom stated this was for fear of negative consequences, even though they deemed this information useful for medical care planning.

Our study showed that in a non-gatekeeping system like Germany, GPs still play a decisive role in caring for lesbian women. In our study sample every second women sees a gynaecologist on a regular basis, mostly for gynaecological prevention, the most frequently accessed preventive service (by 70% of participating women). However, GPs remain the main contact persons for healthcare needs. This corresponds with GPs’ function as coordinator in healthcare, providing a comprehensive and general approach to medicine. The findings highlight a need among GPs to foster a deeper knowledge and awareness of problems associated with homosexuality. There is no doubt that women concealing their sexual preference and GPs ignoring this part of patients’ life risk missing appropriate actions, including preventive services or referrals [32–34]. About 20% of the women in our study reported psychological symptoms as reason for encounter, but bio-psycho-social counselling may not be effective when women avoid disclosing their sexual orientation [35]. In our study more women disclosed to their gynaecologist than to their GP and only 5% were directly asked about their sexual orientation by the physician. It may be that physicians do not recognize the information’s relevance for non-gynaecological health issues and that disclosure to a gynaecologist is easier because it seems to be more obvious to discuss sexual issues in this context of health care than for example with a GP.

There is one other similar study in Germany by Dennert et al. who obtained quantitative data from 578 lesbian women attending a special spring meeting in Cologne. Similar to our study, the authors found that half of the respondents had not informed their primary care providers about their sexual orientation, and indeed preventive measure in the study sample were less frequent than in a representative sample of the gender population [36, 37]. Disclosure is of particular importance in primary care for the detection of healthcare needs and tailoring of treatment. We therefore aimed to identify factors associated with disclosure to GPs: Young age, a higher educational level and those who are not out to other physicians were more likely to reveal their sexual orientation to their primary care physician. We found no significant association between disclosure to primary care providers and the time since outing or previous discrimination experiences. Our results are in line with other studies that confirmed the influence of education as a predictor of disclosure [35]. Additionally, St. Pierre et al. found a positive association between higher income and disclosure of sexual orientation to primary care providers [38]. Other predictors for a higher probability of disclosure from different studies are: less internalized homophobia, a stronger connection to the LGBT community, not having an immigration status, not having a history of a medical condition, a higher provider’s gay-positivity and a higher level of the patient’s outness [10]. All studies show methodological limitations and the included factors overlap only marginally between studies, which impedes the distinction between real effects, confounding or setting specific phenomena.

A positive interpretation of the observed association between young age and disclosure in our study could suggest that societal changes towards less stigmatization and more tolerance favourably affect younger lesbians’ perception of healthcare. In the United States, there has been improved utilization of preventative services among lesbian women, as described by Roberts et al. [39], but a recent report by the European Union Agency for Fundamental Rights (FRA) highlights frequent ongoing discrimination and violence against lesbian, gay, bisexual and transgender individuals in all member states, including Germany [40].

Discrimination of lesbian women in German healthcare has only begun to be studied. We found that 12% of participants did not share their sexual orientation with their GP for fear of negative consequences, even though they deemed this information useful for medical care planning. Twelve percent of participants had also experienced discrimination in the past. The only other study on discrimination experiences in German healthcare found a corresponding rate of 20%, where 20% also did not share their sexual orientation with any physician (any speciality) [37].

Awareness of lesbian health needs and initiatives for improvement seem to fall behind in Germany compared to Anglo-American countries, where position papers and recommendations have been published to advise on special healthcare needs and the avoidance of discriminating behaviour [27, 41]. A comparable statement for Germany does not yet exist, even though there is evidence of perceived barriers to healthcare among lesbian women. When asked for their suggested areas for improvement, women participating in our study named gender neutral language, flyers on homosexuality in the waiting area, involvement of a spouse, training of physicians, a directory of homosexual physicians, and labelling as a lesbian-friendly practice. There have been interventions claiming to alleviate these problems, but to our knowledge there is no evidence showing their effectiveness. Innovative treatment and service programs tailored to address the specific needs of sexual minority groups should be developed, implemented and evaluated [42]. According to Kerr et al., healthcare providers should undergo diversity and sensitivity training to work more effectively with these groups, and physicians should inquire about the sexual orientation of their patients [43].

There are limitations to our study. We applied convenience sampling, which is a debatable method [44], but it is still an acknowledged technique for reaching underrepresented populations [45]. Research in lesbian health is largely based on self-recruited or convenience-based samples. This poses problems regarding the generalisation of results depending on the study’s aim [46]. Our samples (internet based and at special events) may be biased, but the described similarities of study results with national and international studies suggest a reasonable external validity. Convenience-based sampling can produce valuable results [47]. We therefore hope our results will stimulate further research.

We compared internet- and paper-based samples of lesbian women in Germany and found neither statistically significant nor content-relevant differences between the two groups [30]. Data merging therefore seems justifiable. Similar approaches have been chosen by studies in different contexts [48]. When interpreting our healthcare utilization data, one needs to consider that we relied on self-report measures, which tend to overestimate participation rates in preventive health screenings [49], whereas issues concerning mental health (as a sensitive topic) are most likely underestimated [8].

Our findings might be biased giving the socio-demographic profile of our respondents as our sample were predominantly young and well educated.

## Conclusions

We conclude that our findings on lesbian women’s access to healthcare and their expectations towards primary care physicians in Germany highlight the considerable responsibility of the GP (even in a non-gatekeeping healthcare system). It is up to the GPs to create an open atmosphere and to acquire the respective knowledge to provide adequate treatment. Caring for marginal groups should be incorporated in medical training and further education. Furthermore it may be prudent to promote a management approach to healthcare, where physicians address patients’ sexual orientation pro-actively in order to address individual needs accordingly.

## Additional file

Additional file 1: Study Questionnaire. English translation of the items used in the study. (DOCX 84 kb)

## Abbreviations

Df: Degrees of freedom
FRA: European Union Agency for Fundamental Rights
GP: General practitioner
LGBT: Lesbian, gay, bisexual, transgender
PHQ-2: Patient health questionnaire
