# Supplementary material for: Lesbian womens’ access to healthcare, experiences with and expectations towards GPs in German primary care
Source: BMC Fam Pract. 2016 Nov 21;17:162. doi: 10.1186/s12875-016-0562-4 (PMC5117504; doi:10.1186/s12875-016-0562-4)
Supplement: Additional file 1: — Study Questionnaire. English translation of the items used in the study. (DOCX 84 kb) [file 12875_2016_562_MOESM1_ESM.docx]

| 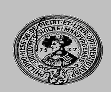 | **Philipps University Marburg**  **Department of General Practice /Family Medicine** Prof. Dr. Annette Becker, MPH Cand. Med. Karina Löltgen [Annette.Becker@staff.uni-marburg.de](mailto:Annette.Becker@staff.uni-marburg.de), Tel.: 06421/28-65120  **Questionnaire  Healthcare provision for**  **lesbian women** | | |
| --- | --- | --- | --- |
| **Thank you very much for your participation!** | | | |
| **Demographics** | | | |
| **1.0 What is your age ?** | | _ _ years | |
| **1.1 Which is your highest secondary education level ?**   - Secondary modern school - Vocational school - Middle school (former GDR) - Advanced technical college / University-entrance diploma - Other degree, which?______________________________________ - No degree | | | |
| **1.2 What kind of professional education do you have?**   - vocational training (in-firm training / vocational training) - educational training (vocational school, business school, technical school) - university / Studies, which?_________________________________ - other professional education - no professional education | | | |
| **1.3 Are you employed?**  Yes,   - full time - part time - less than part time | | | No,   - housewife - in training / retraining - unemployed - pension |
| **1.4 What is your average monthly pre-tax income?**   \| ❑ up to 400 € \| ❑ 400-1000 € \| ❑ 1000-4000 € \| ❑ more than 4000 € \| ❑ no income \| \| --- \| --- \| --- \| --- \| --- \| | | | |
| **1.5 What is your marital status?**   - single - in a relationship - registered relationship - divorced - widowed - other_________________________________ | | | |
| **1.6 Do you live together with your partner?**  ❑ Yes ❑ No | | | |
| **1.7 Are you a parent or a co-parent?**  ❑ Yes ❑ No | | | |
| **1.8 How is your contact to your family of origin (parents or where you have grown up)?**   - no contact - sporadic contact - regular contact - intensive contact | | | |
| **1.9 How is the quality of your contact to your family of origin?**   \| **1** \| **2** \| **3** \| **4** \| **5** \| **6** \| **7** \| **8** \| **9** \| **10** \| \| --- \| --- \| --- \| --- \| --- \| --- \| --- \| --- \| --- \| --- \|   very good very bad | | | |
| **Questions on sexuality** | | | |
| **2.0 How would you describe your sexual behavior?**   \|  \|  \|  \|  \|  \|  \|  \| \| --- \| --- \| --- \| --- \| --- \| --- \| --- \| \| exclusively  heterosexual \| mainly heterosexual,  occasionally homosexual \| mainly heterosexual, more than  occasionally homosexual \| evenly  heterosexual and  homosexual \| mainly homosexual, more than  occasionally heterosexual \| mainly homosexual,  occasionally heterosexual \| exclusively  homosexual \| | | | |
| **2.1 Do you participate in the lesbian community or in lesbian specific events?**   - never - rarely/ irregularly - regularly - often - very often | | | |
| **2.2 Are you out (have you disclosed your sexual orientation)? If yes, to whom?**   - no disclosure - just to close friends - just to family of origin - just to family of origin and friends - comprehensive disclosure (friends, family, colleagues) | | | |
| **2.3 If you are out, do you feel accepted from? (several responses possible)**   - family of origin ❑ not out - friends - colleagues - society | | | |
| **2.4 How long are you out?** ❑ not out  ❑ less than 12 months ❑ 1-5 years ❑ more than 5 years | | | |
| **Questions on health provision** | | | |
| **2.5 What is your health insurance?**   - statutory health insurance (AOK, BKK, ..) - private health insurance ❑ additional health insurance - no insurance (dental prostheses, hospital) | | | |
| **2.6** **Are you on regular medication (most days of the week)?**  **(several responses possible)**   - No - Yes, over-the-counter medication - Yes, prescription drugs | | | |
| **2.7 Who is your main medical contact person in health matters?**   - I have none - General Practitioner/Family Doctor - Neurologist - Psychiatrist - Gynaecologist - other__________________________________________________________ | | | |
| **2.8 How often have you consulted your medical contact person in the past 12 months? (except   dentist)**   - never - 1 - 2 times - 2 - 5 times - more than 5 times | | | |
| **2.9 Have you attended preventive medical checkups in the past 3 years?** **(several responses   possible)**   - No - Yes, ……❑ dentist   ❑ pap smear  ❑ mammography  ❑ bowel cancer screening  ❑ [fecal occult blood test](http://flexikon.doccheck.com/de/Fecal_Occult_Blood_Test)  ❑ other,________________________________________ | | | |
| **3.0** **If you have consulted a physician in the past 12 months, what was the reason for encounter?**   - general conditions, acute illness, e.g. common cold, injury, pain, etc. - chronic conditions, e.g. allergies, diabetes, asthma, etc. - psychiatric disorders, e.g. depression, eating disorders, burn-out, etc. - preventive medical check ups, e.g. gynaecologist, etc. - counselling, e.g. reproductive medicine, diet, contraception, smoking cessation, etc. | | | |
| Questions 3.1 bis 3.5 if you have a medical contact person, otherwise continue with question 3.6 | | | |
| **3.1 Is your current medical contact partner informed about your sexual orientation?**  ❑ Yes ❑ No ❑ I don´t know | | | |
| **3.2 Would you like to out yourself to your physician if there would be an opportunity to do so?**   - Yes ❑ I am out - No - I don´t care | | | |
| **3.3 Is it important for you that your physician is informed about your sexual orientation?**  **(several responses possible)**   - No - Yes, because:❑ it creates a better, more open relationship.   ❑ it enables a better healthcare provision / diagnosis.  ❑ no unnecessary questions are asked.  ❑ no unnecessary examinations / treatments are performed.  ❑ less misunderstandings emerge.  ❑ I want to be honest.  ❑ my partner should be involved in treatment decisions. | | | |
| **3.4 If you are out: How did your physician react when you did out yourself?   (several responses possible)** ❑ not out   - negative:   ❑ ignored my outing  ❑ became sexist and intolerant  ❑ wanted to convince me to become heterosexual  ❑ other___________________________________________   - neutral - positive:   ❑ recommended preventive medical check ups  ❑ recommended protection against sexually transmitted diseases  ❑ asked for problems of integration  ❑ other___________________________________________ | | | |
| **3.5 After that, did your physician forget that you are lesbian and you had to remind him/her or repeat your outing?**   - Yes ❑ No ❑ not out | | | |
| \| **3.6 Which criteria are important for**  **you to choose a physician when**  **you have a health problem?** \| **very**  **impor-tant** \| **important** \| **don´t care** \| **unimpor-tant** \| **very**  **unimpor-tant** \|  \| \| --- \| --- \| --- \| --- \| --- \| --- \| --- \| \| ▼ \| ▼ \| ▼ \| ▼ \| ▼ \|  \| \| **competency / expert knowledge** \| ❑ \| ❑ \| ❑ \| ❑ \| ❑ \|  \| \| **sensibility / sensitiveness** \| ❑ \| ❑ \| ❑ \| ❑ \| ❑ \|  \| \| **open-mindedness towards**  **homosexual individuals** \| ❑ \| ❑ \| ❑ \| ❑ \| ❑ \|  \| \| **gender of physician** \| ❑ \| ❑ \| ❑ \| ❑ \| ❑ \|  \| \| **sexual orientation of physician** \| ❑ \| ❑ \| ❑ \| ❑ \| ❑ \|  \| \| **recommendation from**  **family / friends** \| ❑ \| ❑ \| ❑ \| ❑ \| ❑ \|  \| \| **other_________________________** \| ❑ \| ❑ \| ❑ \| ❑ \| ❑ \|  \| | | | |
| **3.7 Where do you collect your information regarding health matters?**  **(several answers possible)**   - physician - internet - literature / textbooks - friends - health insurance - other______________________________________________________ | | | |
| Experience with the health system | | | |
| **3.8 Do you have a specific demand of medical provision because of your sexuality?**   - No ❑ Yes: ❑ education (e.g. HIV)   ❑ counselling  ❑ psychological support  ❑ assistance / counselling regarding outing  ❑ reproductive medicine  ❑ other___________________________________ | | | |
| **3.9 Do the physicians you are consulting mainly use gender neutral expressions?**   - never ❑ rarely ❑ often ❑ always | | | |
| **4.0 Do you regard the specific medical provision as problematic:**  **Would you like to have a main medical contact person but you don´t find one?**   - Yes ❑ No | | | |
| **4.1 Do you think that the knowledge of your medical contact person regarding homosexuality is  sufficient?**   - Yes, completely - Yes, but it could be better - No, but has basic knowledge - No, has no knowledge - Do not know | | | |
| **4.2 Have you made rather positive experiences with the healthcare system?**  **(several answers possible)**   - Yes: ❑ tolerance   ❑ counselling / support  ❑ recommendation of special groups/information centers  ❑ other__________________________________________________   - I don´t know / Can´t evaluate this - No: ❑ discrimination   ❑ inadequate comments / jokes  ❑ not taken seriously  ❑ other______________________________________ | | | |
| **4.3 Do you feel disadvantaged because of your sexual orientation?**   - Yes ❑ No | | | |
| **4.4 Do you have unmet medical desires? Which?**   - No - Yes ❑ general medical concerns   ❑ provision of information centers  ❑ information on sexually transmitted diseases  ❑ reproductive medicine  ❑ other_____________________________________ | | | |
| **4.5 Have you ever refrained from a necessary examination or treatment because you were   afraid of being discriminated because of your sexual orientation?**  ❑ Yes ❑ No | | | |
| **4.6 Have you ever felt discriminated against by physicians, in hospitals, or in other areas of the  healthcare system because of your sexual orientation**  ❑ Yes ❑ No | | | |
| **4.7 Have you ever been refused an examination or treatment because of your sexual   orientation?**  ❑ Yes ❑ No | | | |
| **4.8 Have you ever felt that your physician should know about your sexual orientation prior to   an examination or treatment, but you did not disclose it for fear of negative consequences?**  ❑ Yes ❑ No | | | |
| **4.9 Did you ever feel the need to talk about your sexual orientation with your medical contact   person, but he/she dismissed it?**  ❑ Yes ❑ No | | | |

| Question regarding your health | | | | | | | | |
| --- | --- | --- | --- | --- | --- | --- | --- | --- |
| **4.10 Over the past 2 weeks, how often have you**  **been bothered by any of the following  problems?** | | Not at all  ▼ | | Several days  ▼ | | More than half the days  ▼ | | Nearly every day  ▼ |
| Little interest or pleasure in doing things | | ❑ | | ❑ | | ❑ | | ❑ |
| Feeling down, depressed or hopeless | | ❑ | | ❑ | | ❑ | | ❑ |
| **4.11** **In the past 4 weeks were you bothered by the  following problems?**  panic attack (sudden feelings of fear or panic) | |  | |  | | **N****O**  ❑ | | **YES**  ❑ |
| nervous tension, fearfulness, feeling of  being out of balance | |  | |  | | **❑** | | **❑** |
| **4.12 How strongly have you been bothered in the past 4 weeks by the following problems?** | Not  impaired | | | | Less  impaired | | Strongly  impaired | |
| abdominal pain | **❑** | | | | **❑** | | **❑** | |
| back pain | **❑** | | | | **❑** | | **❑** | |
| pain in arms, legs oder joints (knee, hip, etc.) | **❑** | | | | **❑** | | **❑** | |
| problems with menstruation | **❑** | | | | **❑** | | **❑** | |
| pain or problems with sexual intercourse | **❑** | | | | **❑** | | **❑** | |
| headache | **❑** | | | | **❑** | | **❑** | |
| chest pain | **❑** | | | | **❑** | | **❑** | |
| dizziness / syncope | **❑** | | | | **❑** | | **❑** | |
| palpitation or tachycardia | **❑** | | | | **❑** | | **❑** | |
| shortness of breath | **❑** | | | | **❑** | | **❑** | |
| constipation, nervous bowel or diarrhoea with  nausea / flatulence | **❑** | | | | **❑** | | **❑** | |
| **4.13 Over the past 2 weeks, how often have you**  **been bothered by any of the following  problems?** | Not at all | | Several days | | | More than half the days | | Nearly every day |
| Difficulties falling asleep, sleeping through or  increased sleep | **❑** | | **❑** | | | **❑** | | **❑** |
| Tiredness or lack of energy | **❑** | | **❑** | | | **❑** | | **❑** |

| **4.14 We would like to improve the healthcare provision for lesbian women.**  **What kind of support do you like to have in health matters? Do you have desires,**  **proposals or needs?**  _____________________________________________________________________________  _____________________________________________________________________________  _____________________________________________________________________________  _____________________________________________________________________________  _____________________________________________________________________________  _____________________________________________________________________________  _____________________________________________________________________________  _____________________________________________________________________________ |
| --- |
| **5.0 How did you get this questionnaire?**  ❑ Internet  ❑ friends  ❑ group / community / information center  ❑ personal contact |
| *****Thank you very much for your participation!*****  You can answer this questionnaire alternatively online or forward the address of the web page to those who might be interested to participate: ___ www.limeservice.de __ |
